# Modal Analysis of Cerebrovascular Effects for Digital Health Integration of Neurostimulation Therapies—A Review of Technology Concepts

**DOI:** 10.3390/brainsci14060591

**Published:** 2024-06-10

**Authors:** Marcel Stefanski, Yashika Arora, Mancheung Cheung, Anirban Dutta

**Affiliations:** 1 School of Engineering, University of Lincoln, Lincoln LN6 7TS, UK; 2 Department of Biomedical Engineering, University at Buffalo, Buffalo, NY 14228, USA

**Keywords:** transcranial electrical stimulation, functional near-infrared spectroscopy, digital health, precision medicine

## Abstract

Transcranial electrical stimulation (tES) is increasingly recognized for its potential to modulate cerebral blood flow (CBF) and evoke cerebrovascular reactivity (CVR), which are crucial in conditions like mild cognitive impairment (MCI) and dementia. This study explores the impact of tES on the neurovascular unit (NVU), employing a physiological modeling approach to simulate the vascular response to electric fields generated by tES. Utilizing the FitzHugh–Nagumo model for neuroelectrical activity, we demonstrate how tES can initiate vascular responses such as vasoconstriction followed by delayed vasodilation in cerebral arterioles, potentially modulated by a combination of local metabolic demands and autonomic regulation (pivotal locus coeruleus). Here, four distinct pathways within the NVU were modeled to reflect the complex interplay between synaptic activity, astrocytic influences, perivascular potassium dynamics, and smooth muscle cell responses. Modal analysis revealed characteristic dynamics of these pathways, suggesting that oscillatory tES may finely tune the vascular tone by modulating the stiffness and elasticity of blood vessel walls, possibly by also impacting endothelial glycocalyx function. The findings underscore the therapeutic potential vis-à-vis blood-brain barrier safety of tES in modulating neurovascular coupling and cognitive function needing the precise modulation of NVU dynamics. This technology review supports the human-in-the-loop integration of tES leveraging digital health technologies for the personalized management of cerebral blood flow, offering new avenues for treating vascular cognitive disorders. Future studies should aim to optimize tES parameters using computational modeling and validate these models in clinical settings, enhancing the understanding of tES in neurovascular health.

## 1. Introduction

Transcranial electrical stimulation (tES) offers a promising approach to evoke CBF [1] to study cerebrovascular reactivity (CVR), which can be partly effected by the somato-autonomic responses to transcutaneous stimulation [2]. Autonomic reflexes play a vital role in maintaining homeostasis as they regulate blood pressure, breathing, and tissue oxygenation. After an autonomic reflex occurs, the autonomic nervous system (ANS) transmits signals from internal organs (such as the vasculature and heart) to the central nervous system (CNS). The vagus nerve is primarily responsible for conveying these signals to regions like the medulla, pons, and hypothalamus, including during the orienting reflex to a change in the environment. The noradrenergic (NA) nucleus locus coeruleus (LC) is a brain region that becomes activated in parallel with the autonomic nervous system [3]. The LC is a brainstem nucleus involved in arousal, attention, and responses to novel stimuli. LC-NA responds to biological imperatives, which can be either spontaneous reactions to unexpected salient or threatening stimuli, or conditioned responses to anticipated behaviorally relevant stimuli. So, the activation of the LC can be associated with the general orienting reflex [4]; when we encounter something new or unexpected, the LC becomes active, helping us focus our attention and respond appropriately. However, the role of this autonomic reflex including LC-NA in regulating CBF is not fully understood, and the contribution of the baroreflex in CBF regulation is debated [5]. Recent studies in animal models and the mathematical analysis of cardiovascular signals in humans provided insights into the interplay between the arterial baroreceptor reflex (baroreflex) and arousal [6]. Mild baroreceptor stimulation, especially under anesthesia, may inhibit cortical arousal, while significant increases or decreases in baroreflex activation induce arousal in both animals and humans under normal physiological conditions. Additionally, cardiovascular changes during autonomic arousals and transitions between wakefulness and sleep involve adjustments in the baroreflex set point and its equilibrium with central autonomic commands. While acute increases in systemic blood pressure trigger peripheral vasodilation via the baroreflex, cerebral vasculature may need to constrict to protect the blood–brain barrier [5]. Here, regional differences in the autonomic outflow between systemic and cerebral blood vessels are possible. Additionally, the impact of direct (autonomic regulation) and indirect (systemic blood pressure regulation) baroreflex influences on CBF may vary [7]. In hypertensive patients, sympathetic nerve activation leads to cerebral vasoconstriction, unlike in normotensive individuals [8]. The complex interplay of the arterial baroreflex with other factors like the cardiac output, respiratory chemoreflex, and physiological conditions in comorbid cardiovascular disease complicates understanding the CBF regulation and CVR to tES. Despite these challenges, the autonomic regulation including the LC-NA system response and cardiac baroreflex’s role in maintaining adequate CBF should be acknowledged and investigated vis-à-vis CVR dysfunction, for example, in mild cognitive impairments (MCI) [9] that increase the risk of progression to dementia [10]. Here, LC degenerates early during Alzheimer’s disease (AD), and the loss of norepinephrine may contribute to AD pathogenesis [11]. Aside from neurons, LC terminals densely innervate brain intraparenchymal arterioles/capillaries, and norepinephrine modulates astrocyte functions. The neurovascular unit (NVU), which involves interactions between neurons, glial cells, and brain vessels, plays a fundamental role in coupling the energy demand with the regional cerebral blood flow. It includes the blood–brain barrier (BBB) and participates in neuroinflammation and the glymphatic system. NVU alteration is implicated in AD pathophysiology due to relative oligemia in activated brain regions and impaired BBB integrity, contributing to the intracerebral accumulation of insoluble amyloid. The interaction between LC and NVU in AD pathogenesis remains an area of study with therapeutic tES applications [2].

We presented a physiological modeling approach [12] based on the physiology of neurovascular tissues for assessing the vascular response to electric fields generated by tES through various pathways in NVU. The studies [2] presented a physiological model that incorporated the NVU components, including a vascular smooth muscle, perivascular space, synaptic space, and astrocyte cell. The model aimed to capture the effects of transcranial electrical stimulation (tES), specifically transcranial alternating current stimulation (tACS), on direct and indirect vascular responses. Four nested NVU compartmental pathways were proposed, allowing the simulation of tES-induced vessel volume responses. In computational analysis, the tES current density, acting as an input pulse, perturbed state variables in each NVU compartment. This study considered four simulated pathways for vessel response modulation: synaptic potassium, astrocytic membrane potential, perivascular potassium currents, and voltage-gated ion channels on smooth muscle cells. These pathways were designed to simulate vessel oscillations within the frequency range <0.2 Hz. Modal analysis [13,14], a technique commonly used in structural and fluid mechanics [14], was applied to derive the characteristic dynamics of the NVU model. Modal analysis involves determining the system’s natural frequencies and damping factors, allowing the development of a mathematical model describing the system’s behavior. While modal analysis is traditionally used in engineering fields, the study applied this approach to analyze the NVU model, specifically focusing on evaluating neurovascular coupling modes induced by tACS. Then, the design of the controls is imperative to modify the natural behavior of interconnected synchronous generators in NVU systems. Despite the inherent nonlinearity of NVU systems, accurately predicting oscillations around an operating point is possible through linearized system models [12], which justifies the application of linear control theory [15] for the design of tES controllers.

The examination of tES effects, both immediate and prolonged, and the design of controllers is an area of interest of neuroscientific research [12,16,17]. We examined an fMRI-tES dataset [18] with a TR of 3.36 s, revealing a similar finite impulse response hemodynamic response function “FIR HRF” model (with RH, TTP, and FWHM using the rsHRF toolbox [19]) in anodal and sham tDCS conditions at the electrode locations FC5 and PZ ROIs, but differing in the tDCS electrode location FP2 ROI. This discrepancy may be linked to local cortical inhibitory circuits, perivascular nerves, and astrocyte stimulation [18]. Here, prior computational analyses proposed direct perivascular nerve and astrocyte stimulation during tDCS onset leading to an “initial dip” [18]. Prior findings also indicate perivascular space changes (https://www.ismrm.org/workshops/2022/Neuromodulation/program.php; accessed on 12 May 2024), which indicated vascular effects but may also raise safety concerns especially for higher intensity 4 mA stimulation. Then, tES is postulated to also impact blood–brain barrier permeability in NVU, influencing the neuronal function [20], which also raises safety concerns. Here, tDCS and tACS differ in their current profiles, with different therapeutic and safety implications. Indeed, short-duration tDCS can have physiological effects, impacting the autonomic and hemodynamic response [2]. Then, tES sympathoexcitation, likely from somato-autonomic reflexes, reflected in pupil dilation [2], may impact glucose regulation via noradrenaline’s impact on cellular energy processes [21] and neurometabolic coupling. Here, fNIRS’s total hemoglobin (blood volume) changes due to neurovascular and neurometabolic coupling needs further investigation vis-à-vis their correlation with pupil dilation [2]. In animal studies, the pupil diameter has been shown to be inversely correlated with cortical hemodynamics during rest (and non-REM sleep), while when the mouse is alert, moving, or stimulated (as well as during REM sleep), positive correlations were found between the pupil diameter and blood volume, possibly linked to the bistability [22] of norepinephrine-linked astrocytic activity [23]. Therefore, the correlation analysis of pupil dilation vis-à-vis fNIRS’s total hemoglobin (blood volume) changes may delineate tES effects via LC-NA [24]. Also, a mechanistic understanding of the glucose–neurovascular tissue interaction during tES is crucial [2]. Here, the tES sympathoexcitation (indirect effect) and direct modulation of the membrane potential of smooth muscle cells [12], particularly with specific oscillatory frequencies, is examined via frequency domain modal analysis [2].

It is postulated that orthosympathetic activation can affect pial and perforant arteries (>0.05 Hz), while LC-NA affects intraparenchymal arterioles and capillaries (<0.05 Hz) [25]. Understanding these frequency domain modulatory effects of tES on blood vessels necessitates exploring multiple pathways within the NVU. Unravelling these signaling pathways is crucial for comprehending the tES effects on neurons and blood vessels to develop therapeutics, as discussed by Arora et al. [12]. The stimulation-evoked CBF changes depend on multiple pathways including the sympathetic vascular tone (stimulus-related norepinephrine released from sympathetic efferent nerves modulating the vascular tone [26]), against which the neurovascular coupling need to act to dilate the blood vessels [2]. Indeed, it has been shown that the long-term administration of the noradrenaline (norepinephrine) reuptake inhibitor reboxetine (RBX) extended the effects of anodal tDCS on long-term potentiation-like plasticity for over 24 h [27]. RBX also transformed cathodal tDCS-induced long-term depression-like plasticity into facilitation for 120 min. Here, tES effects on autonomic innervation [2] and the activation of noradrenergic receptors can have stimulatory effects on both energy-requiring and energy-yielding processes [21]. Specifically, tES electric field distribution [28] and its activating function [2] can affect the three nerve arrangements in the human cerebral arteries [29]: (a) paravascular nerve bundles outside the tunica adventitia; (b) a meshwork-like perivascular plexus in the outer or middle adventitial zone; and (c) a deep intrinsic perivascular plexus at the adventitial–medial border, oriented transversely. Then, different subtypes of adrenoceptors often activate distinct processes, and the stimulation can allow for the simultaneous enhancement of the oxidative metabolism and/or glycogenolysis, e.g., beneficial in type 2 diabetes [2]. Unlike classical mechanisms, tES effects may enable the stimulation of the energy metabolism without preceding decreases in ATP (for activating ATP-sensitive potassium channels) thereby leading to beneficial effects via the relationship between the metabolism, excitability, and Aβ pathology [30]. The stimulation of glycogenolysis is noteworthy, as it is considered an integral part of glucose breakdown via a significant ‘glucose-glycogen shunt.’ While an increase in mitochondrial Ca^2+^ has been observed in astrocytes, the direct stimulation of the oxidative metabolism by elevated intracellular calcium has been extensively studied, mainly in muscle and the liver. This includes the direct stimulation of mitochondrial dehydrogenases and oxidative phosphorylation, contributing to the understanding of noradrenaline’s impact on cellular energy processes [21].

**Figure 1:**
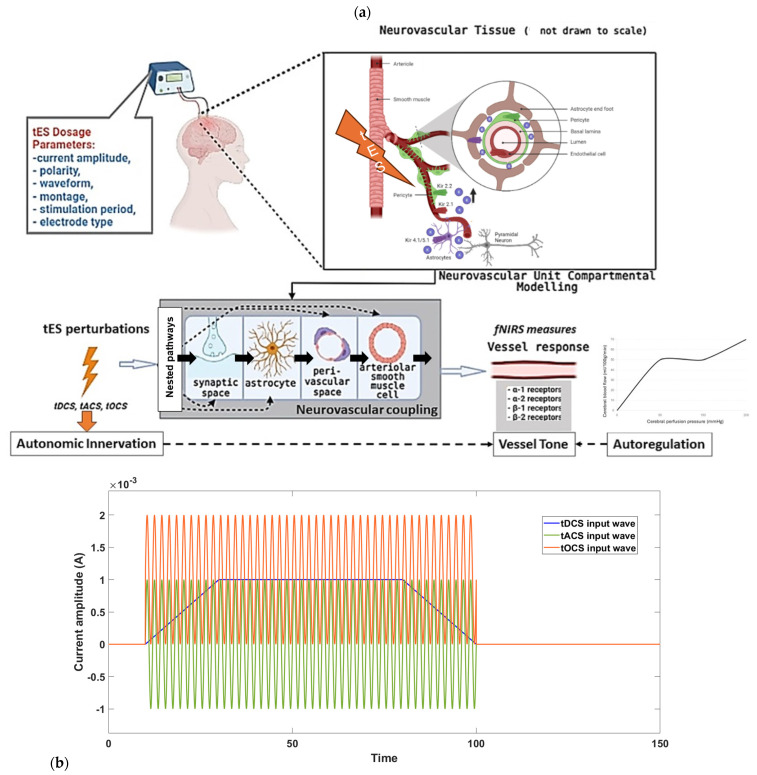
(**a**) Effect of tES dosage on neurovascular tissue: physiological modeling depiction. (**b**) tES time series perturbations for model evaluation. tDCS has a monophasic, non-oscillating baseline, while tACS rhythmically reverses electron flow. Additional methods include tOCS, guiding oscillations with a direct component, and tRNS, injecting bounded stochastic alternating current.

Indeed, neurovascular coupling itself may be tES-modulated [21,31] since the noradrenaline release from LC axons induces vessel tone in arteriolar smooth muscle and contractile capillary pericytes [32], in addition to small tES field-induced smooth muscle cell membrane potential hyperpolarization [33]. Vessel tone is crucial since it enables neuronal activity to trigger vasodilation via neurovascular coupling [32], enhancing local cerebral blood flow regulation—see Figure 1a. In the brain, a significant portion of vascular resistance is in the capillaries, and LC axons release noradrenaline closer to pericytes than to arterioles. Then, the cerebral adrenoreceptor distribution exhibits heterogeneity, indicating the region-specific autonomic regulation of CBF [34]. Cerebral circulation features unique compensatory responses, involving chemo- and autoregulatory mechanisms, and interactions with parasympathetic nerve activity. This interplay between sympathetic and parasympathetic reflexes ensures the optimal perfusion of CBF in response to changing perfusion pressures, aiming to optimize oxygen and nutrient delivery to the brain while maintaining the blood volume and intracranial pressure. Here, tES effects on the autonomic innervation of the cranial circulation from the external noradrenergic innervation can include a predominant sympathetic component from the superior cervical ganglion and a cranial parasympathetic component that passes through the pterygopalatine (sphenopalatine) and otic ganglion [11]. Claassen and colleagues [35] discuss the mechanisms contributing to myogenic responses, focusing on the regulation of vascular tone, intravascular pressure distribution in the brain, and the autoregulation of CBF. Also, factors include the sensitivity of the vascular diameter to tES changes in the cellular membrane potential, influenced by voltage-dependent calcium channels (e.g., CaV2.1) and large conductance potassium channels (BK_Ca_) [36]. Then, mechanotransducers are proposed as sensors for pressure changes leading to the depolarization of vascular muscle and increased intracellular Ca^2+^. This, in turn, activates contractile proteins, resulting in vasoconstriction. Also, the local release of Ca^2+^ can activate BK_Ca_, inducing hyperpolarization and limiting vasoconstriction [36]. Therefore, the autonomic modulation of the vascular tone needs to be well-coordinated with the neurovascular coupling-related effects for an adequate CBF response to quick task-related metabolic needs (this may be compromised in type 2 diabetes [37]) that may be facilitated with tES, e.g., burst tES [38].

In individuals over 60 years of age, tDCS has been shown to induce cardiovascular and autonomic improvements, enhancing ventricular repolarization dispersion timing, decreasing sympathetic activity and peripheral resistance, and increasing vagal sinus activity and baroreflex sensitivity [39]. Although the authors [39] hypothesized that the anodal tDCS effects on the neuronal networks in the temporal cortex and insular cortex are linked to autonomic nervous system regulation and the perception of emotional sensations within the body, they did not compare the effects of anodal tDCS at an unrelated brain area and so the mechanism of action (e.g., autonomic reflex arc) is unclear. Another study [40] investigated the impact of repetitive tDCS on cerebral perfusion—anodal left prefrontal tDCS was administered over three consecutive days, leading to widespread increases in perfusion, suggesting a heightened metabolism. In contrast, a matched group receiving sham tDCS showed general perfusion decreases. The active stimulation group exhibited significantly greater perfusion increases in various brain areas, particularly in the LC and neocortex regions associated with object recognition and attentional modulation. The observed changes in the neocortex may result directly from stimulation or indirectly through altered noradrenergic system activity from LC modulation, which can also modulate neurovascular coupling [31]. Another study [24] suggests that the effects of electrical stimulation, specifically occipital nerve tDCS (ON-tDCS), involve ascending fibers of the occipital nerve synapsing with neurons in the nucleus tractus solitarius. These neurons then project to the LC, promoting NA release and enhancing functional connectivity with the hippocampus. Here, we suggest a short-duration ON-OFF tDCS time series (see Figure 1 in [41]) acting as slow transcranial oscillating-current stimulation (tOCS) [2] leveraging tissue low-pass filtering which may act through superficial nerves, noradrenergic axons, and efferent innervation to evoke a beneficial mural cell response for neurovascular coupling [42]. Unlike pharmaceutical approaches, ON-tDCS offers the potential for persistent, stimulus-specific changes to neural circuits with minimal side effects. Here, tES under portable brain imaging [17] could be an individualized therapy option for conditions involving insufficient LC-NA function [11], including late-life depression leading to mild cognitive impairment [43,44], where the effects may be mediated by the individual’s cerebrovascular and NVU status.

## 2. Materials and Methods

The major factors involved in the design of tES dosage are: current amplitude, waveform, polarity, duration, montage, and electrode specifications [45], as depicted in Figure 1b. These factors are crucial in neuromodulating specific characteristics. For instance, tOCS has been shown to facilitate corticospinal excitability phase independently, both on- and off-line, similar to tDCS [46]. Meanwhile, tACS was more likely to entrain neuronal activity while blocking sensory input [47]. To comprehend the mechanistic aspects of tES techniques on hemodynamics, we used mathematical model [12] based on the physiology of neurovascular tissue for evaluating the vascular response through various paths that are susceptible to the electric fields generated by tES, as shown in Figure 1a. Our simulation model was constructed with four compartments, drawing from the existing literature: synaptic space, astrocyte space, perivascular space, and arteriole smooth muscle cell space. To simulate the vessel volume response within the physiological model, we designed four nested neurovascular unit (NVU) compartmental pathways. Each state variable in these pathways could be influenced by the transcranial electrical stimulation (tES) current density, acting as the input pulse. The simulations of the model considered different tES-induced perturbations: synaptic potassium release from active neurons for Pathway 1, astrocytic transmembrane current for Pathway 2, perivascular potassium concentration for Pathway 3, and voltage-gated ion channel current on the smooth muscle cells (SMC) for Pathway 4. Detailed information regarding the model implementation and computational analysis can be found in publication [12].

Physiologically detailed models [12] were executed using the ‘ode23tb’ solver in Simulink Release 2019b (MathWorks, Inc., Natick, MA, USA). These models simulated oscillations ranging from 0 to 0.2 Hz in response to tDCS perturbations. Subsequently, we subjected the four nested NVU compartmental pathways [12] to perturbations from transcranial oscillating current stimulation (tOCS), tDCS, and transcranial alternating current stimulation (tACS) at varying frequencies (0.1 Hz to 10 Hz), conducting a sensitivity analysis for blood vessel diameter changes. This comprehensive approach considered the vascular effects of tES, incorporating both neuronal and non-neuronal mechanisms with distinct sensitivity levels. Notably, within the frequency range of 0.1 Hz to 10 Hz, we observed that vessel oscillations exhibited greater sensitivity to tOCS than to tACS, and entrainment effects were more pronounced at lower frequencies. Subsequently, modal analysis was performed on the physiological model where we applied ten random tES perturbations, utilizing bandpass-filtered (0.01–1 Hz) white noise inputs, to the four implemented physiologically detailed models [12]. The input and output time series were recorded using the time-domain data object (‘iddata’ in MATLAB Release 2019b, MathWorks, Inc., USA). For modal analysis, we focused on the oscillatory component of the vessel response, excluding the initial 50 s of time series data. Modal analysis functions, including ‘modalfrf,’ ‘modalfit,’ and ‘modalsd,’ were applied to the data object to generate frequency-response functions, natural frequencies, and stabilization diagrams, respectively. The natural frequencies of the four system modes, determined from the measured frequency-response functions (frf) at frequencies (f) and a sample rate of 10 samples per second, were calculated using the peak-picking method. The peak-picking method posits that each notable peak in the frf represents a single natural mode; however, this does not ensure the orthogonality of the modes in a multi-degree of freedom (MDOF) system of nxn matrices such that it can be more simply described as a series of *n* decoupled single-degree of freedom (SDOF) system. The natural modes of a discrete linear system exhibit orthogonality in relation to the mass and stiffness matrices in case of structural mechanics, but this orthogonality is understood in a generalized form. Generally, natural modes are not orthogonal in the usual sense, meaning the dot product of two modal vectors typically is not zero. However, certain simple conditions can be introduced to achieve ordinary orthogonality of these modes in fluid mechanics [48] to address the loss of orthogonality issue. Then, ‘modalsd’ estimates the natural frequencies and damping ratios and generates the stabilization diagram using the least-squares complex exponential method that calculates the impulse response for each frequency-response function and fits it with a series of complex damped sinusoids using Prony’s method.

Figure 1a outlines proposed mechanism through which tES influences the perivascular space, as theorized by Arora et al. [12]. The hypothesis posits that tES modulates the vasculature via the perivascular pathway (including autonomic innervation and activation of noradrenergic receptors—see Figure 1a), leading to vasoconstriction increasing and vasodilation decreasing the perivascular space volume. In a computational model [12], an immediate vascular response was captured through the perivascular pathway, involving the interaction between perivascular potassium and calcium concentrations, resulting in steady-state vessel oscillations below 0.1 Hz. These oscillations may potentially phase synchronize with neuronal oscillations, motivating the exploration of neurovascular coupling through joint imaging with fNIRS-EEG [17]. Acute tES within a short duration (<150 s), as indicated by Arora et al. [12], can impact the vasculature for the immediate control of blood vessel response using model predictive control (MPC) [2]. Then, the cerebrospinal fluid (CSF) circulation involves two distinguishable components: bulk flow (circulation) and pulsatile flow (back and forth motion) that can be measured using near-infrared transillumination-backscattering sounding methods [49]. The pulsatile flow in CSF is influenced by both cardiac and respiratory cycles reflected in the fNIRS oscillations [50] and there is growing interest in the arterial pulsations driving CSF flow [51,52] that needs model-based analysis [2] of the role of neurovascular coupling [53,54] via synaptic-like transmission between neural axons and arteriolar smooth muscle cells [55]. MPC employs an internal model that considers cortical activity, local metabolic factors, and vascular response to optimize tES control actions over a predefined prediction horizon, operating in a receding horizon fashion for online operation. Then, rapid vascular response observed at the onset of tES [12] could modulate bulk flow of cerebrospinal fluid [56] when applied as slow oscillations according to the resonance frequencies creating an opportunity for human-in-the-loop optimization based on blood volume (total hemoglobin) feedback from fNIRS [2]. Previous research [2] utilizing modal analysis and a case study in a healthy human suggested that an “optimal” oscillatory frequency warrants further investigation on the effects of bulk flow of cerebrospinal fluid [56] vis-à-vis the state of the astrocytes, extracellular glucose [57], and interstitial potassium (tES modulation of neurovascular coupling [42]). 

Our recent findings from Minager et al. (https://www.ismrm.org/workshops/2022/Neuromodulation/program.php, accessed on 12 May 2024) provide support for the immediate alteration of perivascular space morphology by tES that may be linked to the vascular response [2,15]. In a recent magnetic resonance imaging (MRI) study [52], the effects of low-frequency hemodynamics (0.01–0.1 Hz) on cerebrospinal fluid (CSF) movement were explored, with a focus on the correlation between vascular low-frequency oscillations (LFOs) in the neck and CSF directed toward the spinal canal. This study established a temporal relationship between the internal carotid artery and caudally directed CSF movement. The origin of vascular LFOs remained unclear, with potential sources including blood CO2 variations and vasomotion. Additionally, the study investigated the influence of respiration on CSF dynamics, revealing a sequential and continual impact on the CSF-venous system. Cross-frequency coupling suggested that respiration affects CSF dynamics through both pressure-related venous return changes and blood gas-related vessel volume changes. Although low-frequency hemodynamics and respiration contributed to CSF flow, power spectrum analysis indicated that the LFOs play a more substantial role in regulating CSF dynamics than respiration. Cardiac pulsations were found to have a comparatively weaker influence on CSF movement. With the convenience of fNIRS neuroimaging [58] and near-infrared transillumination-backscattering sounding methods [49] in a point-of-care setting compared to MRI [52], the portable neuroimaging-based Model Predictive Control (MPC) of tES [2] is proposed to be applied during sleep to facilitate glymphatic clearance [59] in MCI to protect from AD. Then, decreased metabolite clearance [60] due to sleep deprivation can lead to decreased neurovascular coupling responses [61]; however, alterations in functional connectivity due to sleep deprivation are likely attributable to impaired transitions within brain networks during task performance, possibly related to neurovascular coupling status of different brain regions. To gain a comprehensive understanding of the mechanisms behind sleep deprivation-induced lack in neurovascular homeostasis [62] and their impact on cognitive performance in real-life situations, further longitudinal research is essential [61]. Here, our innovative approach enables the optimization of tES patterns at the point of care to elicit the optimal blood volume response and CSF dynamics, potentially reducing risks associated with heightened metabolic demand with neuronal activation in pathological tissues, such as ischemia in vascular dementia. 

Yashika et al. [12] employed mathematical modeling and hypothesis testing to investigate systems dynamics within four compartments of the neurovascular unit (NVU): Pathway 1—synaptic space, Pathway 2—intracellular astrocyte space, Pathway 3—perivascular space, and Pathway 4—intracellular space of the arteriolar smooth muscle cells (aSMCs). A system identification approach using a physiologically constrained linear model was used to analyze NIRS-based CVR measured during anodal high-definition tDCS in healthy individuals. Yashika et al. [12] found that the perivascular Pathway 3 exhibited the best fit with lowest mean square error and Akaike information criterion within the NVU as the primary mechanism for transient CVR at the onset of tDCS. Here, the “initial dip” or vasoconstriction at the start of the anodal tDCS was postulated to be related to afferent stimulation of nerves from the autonomic and sensory ganglia [12]. Researchers debated whether perivascular nerves directly signal aSMCs in Pathway 4 via neurotransmitters and the consensus leaned towards vasodilation resulting from molecules produced by neural activity, often by astrocytes, rather than direct neurotransmission as modeled computationally [12,42]. Recently, Zhang et al. [55] investigated direct neuron–vessel interactions in mice and found that the gaps in astrocyte end feet revealed dendrites and axons forming neuromuscular junctions with aSMCs. Specifically, a single glutamatergic axon dilated the arterioles they innervate through synaptic-like transmission at neural–aSMC junctions. The presynaptic bouton connected with both postsynaptic dendrites and aSMCs, and these aSMCs expressed various neuromediator receptors, including a low level of the glutamate NMDA receptor subunit 1 [55]. Here, following “initial dip,” the vasodilation due to tDCS [12] can be driven by various neuromediator receptors including aSMCs NMDA receptor for tDCS after effects [63]. Therefore, we augmented Pathway 4 model [12] to include direct neural–aSMC interactions for disentangling the ‘onset response’ of tES [18]. We hypothesize that following “initial dip” or vasoconstriction from pial arterial vasculature downstream to penetrating arterioles, an upstream vasodilation from aSMCs of penetrating arterioles and first-order vessels due to tES needs investigation. Here, under the assumption of expression of glutamate receptors in human aSMCs, Pathway 4 [12] can capture perivascular glutamatergic signaling at the neurovascular junctions. Then, the activation of NMDA receptors in neurovascular junctions is postulated to trigger the influx of calcium ions that bind to and activate BK channels, leading to an increased potassium ion efflux. This process induces membrane hyperpolarization, ultimately causing the relaxation of aSMCs and upstream penetrating arterioles. So, tES electric field perturbs BK current ($\frac{K_{4}}{s/\tau +1}I_{tdcs},$ K_4_ constant, τ time constant) that was added to other currents including I_L_, I_K_, I_Ca_, and I_KIR_ that represent leak, K^+^, Ca^2+^, and KIR channel currents, respectively, in the aSMC compartment from prior work [12]. Then, aSMC membrane potential, V_SMC_, is given:

$$\frac{dV_{SMC}}{dt}=\frac{1}{C_{SMC}}\left(-I_{L}-I_{K}-I_{Ca}-I_{KIR}-I_{KV}+\frac{K_{4}}{s/\tau +1}I_{tdcs}\right)$$

**Figure 2:**
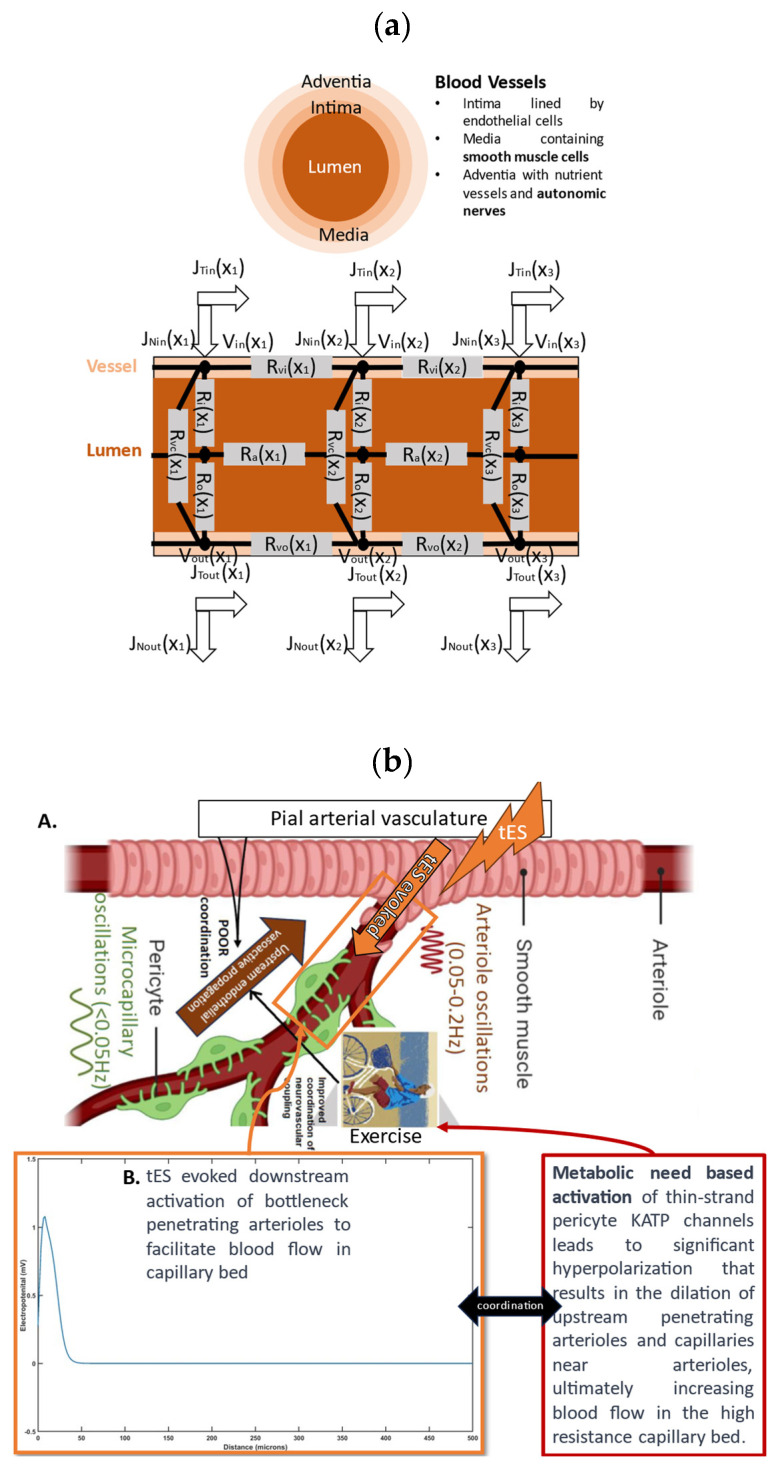

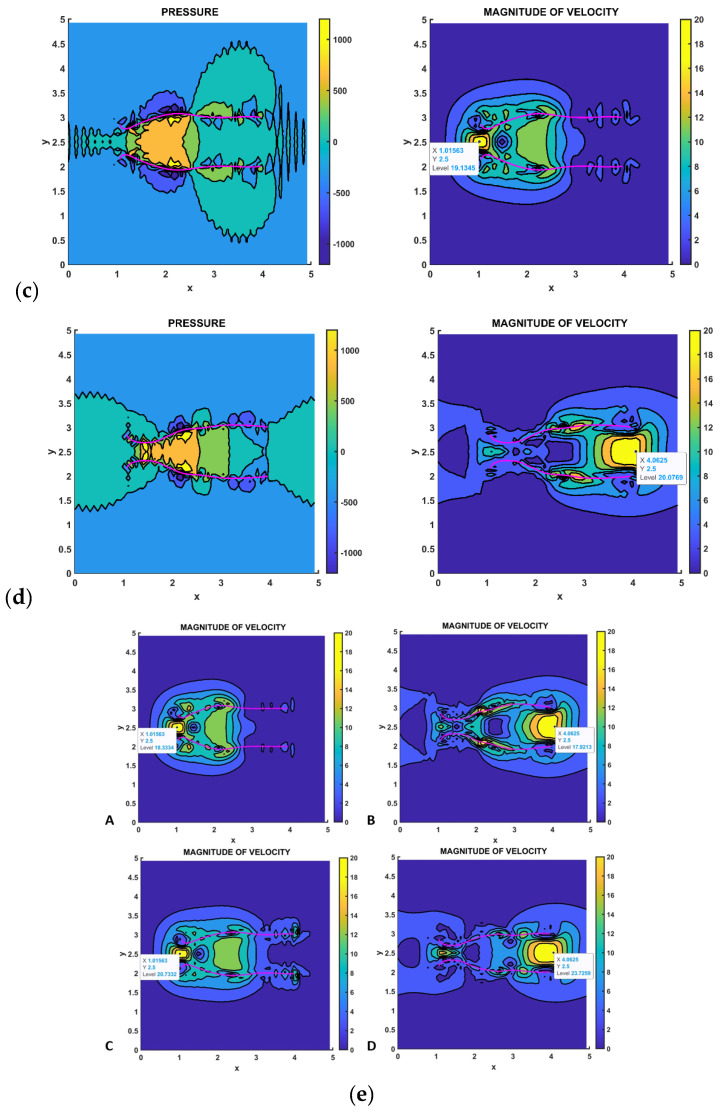
(**a**) The picture displays the transcranial electrical stimulation (tES) voltage at the top (Vin(X1), Vin(X2), Vin(X3)) and bottom (Vout(X1), Vout(X2), Vout(X3)) adventitia layer of a blood vessel’s axial cross-section, along with JT and JN denoting tangential current and R representing lumped electrical resistances in the ohmic model. Investigation of the fluid–structure interaction due to smooth muscle cell’s tES activation with altered modulus of elasticity of bending effects among three consecutive Lagrangian points (X1, X2, and X3) along the penetrating arteriole using a noninvariant beam connecting these nodes. (**b**) We propose that tES initially triggers vasoconstriction in the pial arterial vasculature, prompting blood flow downstream towards the penetrating arteriole—see panel A. Subsequently, tES-induced metabolic demands in the capillary bed activate the upstream penetrating arteriole within the brain parenchyma, resulting in delayed vasodilation—see panel B. We investigated the voltage perturbation required to activate the perivascular nerve using the FitzHugh–Nagumo model along the adventitia layer of the penetrating arteriole (i.e., the bottleneck [66]). The distribution of pressure and velocity in a 300-micron length of the penetrating arteriole in response to perivascular nerve stimulation over a duration of 0.01 s is shown starting from (**c**) to ending in (**d**) due to peristaltic vessel wall motion (data cursor ‘Level’ shows the values). The stimulation begins at the interface to pial arterial vasculature (near cortical surface) at X = 100 microns and extends to X = 400 microns into the brain parenchyma over a duration of 0.01 s, corresponding to a flow speed of 30 mm/s. Here, the arteriole’s modulus of elasticity is 200 million Pascals. (**e**) Distribution of lumen pressure and velocity resulting from peristalsis induced by perivascular nerve stimulation in the vessel wall, illustrated under two different arteriole’s modulus of elasticity. In (**e**), the first scenario (panels A,B) spans from time t = 0 to t = 0.01 s with arteriole’s modulus of elasticity at 0.5 × 2 × 10^8^ Pa. The second scenario (panels C,D) also covers from time t = 0 to t = 0.01 s, but with arteriole’s modulus of elasticity at 2 × 2 × 10^8^ Pa. (data cursor ‘Level’ shows the values).

Temporal interference stimulation (tIS) as well as deep transcranial magnetic stimulation (TMS) can target deep brain regions [64]. Neuronal effects of tIS and TMS can be captured with FitzHugh–Nagumo model [65] that was used to model perivascular nerves of the penetrating arterioles—the bottleneck of neocortex perfusion [66]. For tES, we employ the FitzHugh–Nagumo model to simulate neuronal activity using a sub-threshold stimulus, meaning the stimulus is not strong enough to trigger action potentials (spikes). The microvascular bed consists of specific territories, each supplied by a single penetrating arteriole [66], so facilitating blood flow in the penetrating arterioles with tES/TMS can significantly improve perfusion. Based on prior work [67], tES/TMS-driven perfusion was modeled with an open source fluid–structure interaction software based on Peskin’s Immersed Boundary Method [68]. Since complete blockade of voltage-dependent sodium channels eliminated the excitability changes [63,69], we hypothesize tES modulation of the fast dynamics (sodium current) of the FitzHugh–Nagumo model. In the default model from [68], diffusion coefficient = 10, threshold potential = 0.3, resetting rate = 1, blocking strength = 0.001, and activation strength = 0.05. Then, a Force–Length–Velocity smooth muscle model [68] was used with maximum isometric force produced at the optimum length of the muscle fibers = 100,000, length of the muscle fibers = 1, length at which the muscle fibers exert their maximum tension = 1, and constant specific for each muscle = 0.3. Vascular stiffness was represented by a damped spring mode [68] where a frictional damping force is assumed to be proportional to the velocity of the oscillation that we neglected. Here, we changed the modulus of elasticity from 0.5 × 2 × 10^8^ to 2 × 2 × 10^8^ Pa to study the effects of bending among three consecutive Lagrangian points, such as X1, X2, and X3 (see Figure 2a), employing a noninvariant beam that links these three successive nodes [68]. Figure 2a also shows tES voltage at the top (V_in_(X_1_), V_in_(X_2_), V_in_(X_3_)) and the bottom (V_out_(X_1_), V_out_(X_2_), V_out_(X_3_)) adventitia layer (with nerves) of the axial cross-section of the blood vessel. The J_T_ and J_N_ denote the tangential current at those locations and all R denote the lumped electrical resistances of the ohmic model. 

## 3. Results

Figure 2b shows how pial tES can facilitate downstream blood flow. Figure 2b shows the hypothesis that tES “initial dip” or vasoconstriction from the pial arterial to the penetrating arteriole can force blood downstream, while the tES metabolic need-based upstream activation [70] of the penetrating arteriole leads to delayed vasodilation. Figure 2b shows the tES-evoked electropotential (mV) of the perivascular nerve of a simulated 500-micron-long mesoscopic penetrating arteriole [66] with a 100-micron diameter. Then, a mesoscopic 300-micron-long penetrating arteriole segment with a 100-micron diameter was simulated for fluid–structure–nerve interactions using the FitzHugh–Nagumo neuron model with tES-evoked V_in_(X_1_ = 100 μm) = 1.08 mV near the cortical surface and V_in_(X_2_ = 250 μm) = V_in_(X_3_ = 400 μm) = 0 downstream. Figure 2c shows a FitzHugh–Nagumo model of the penetrating arteriole segment with V_in_(X_1_ = 100 μm) = 1.08 mV at the pial-penetrating arteriole neck region, then V_in_(X_2_ = 250 μm) = V_in_(X_3_ = 400 μm) = 0 downstream. Also, vasodilation takes place downstream from the penetrating arteriole (X_2_ = 250 μm) and roughly to the first-order vessel depth (X_3_ = 400 μm), which are all represented by a single mesoscopic penetrating arteriole shown in pink in Figure 2c,d. The pressure and velocity magnitude in the lumen of the mesoscopic penetrating arteriole due to vasoconstriction (“initial dip”) are shown in Figure 2c where the initially flow velocity is high near X = 100 μm (Figure 2c) and moves downstream to X = 400 μm (Figure 2d) in 0.01 s due to the peristaltic vessel wall motion which is important for ‘coordination’ with the upstream vasodilation— see the hypothesis in Figure 2b. The pressure and velocity magnitude in the penetrating arteriole with peristaltic vessel wall motion for two different moduli of elasticity, 0.5 × 2 × 10^8^ Pa (Figure 2e(A,B)) to 2 × 2 × 10^8^ Pa, are shown in Figure 2e(C,D). Initially, the flow velocity is high, near X = 100 μm (Figure 2e(A,C)), which moves downstream to X = 400 μm (Figure 2e(B,D)) in 0.01 s due to the peristaltic motion (hypothesized coordination with the upstream vasodilation—see Figure 2b). There is an increase in the flow velocity magnitude with an increase in the moduli of elasticity, which can lead to downstream turbulence affecting the endothelial and glycocalyx function [67].

In our conceptual proposition on tES for digital health integration at the point-of-care settings, we augmented the model for Pathway 4 [12] that was motivated by Zhang et al. [55]. We explored the impact of tES on perivascular axons through synaptic-like transmission at neural–aSMC junctions, leading to changes in the vessel circumference. The Pathway 4 model [12] was augmented with tES effects via the FitzHugh–Nagumo model of the perivascular axon at neural–aSMC junctions. This is relevant in vascular cognitive impairments where tES effects on the bottleneck penetrating arterioles may be therapeutic, which can be mediated by vessel stiffness in SVD. Increased vascular stiffness can also be due to elevated glucose levels in diabetes that lead to structural and functional changes in proteins and the implications of protein aggregation and advanced glycation end products [37] in the context of neurovascular coupling in dementia [25]. Indeed, vessel stiffness emerges as a crucial factor in determining hemodynamic oscillations that can be probed using modal analysis [37]. Then, the human-in-the-loop optimization of tES/TMS under NIRS imaging may individualize the dose-response by supporting higher oxygen availability in the capillary bed and paravascular waste clearance [2] via the optimal peristaltic motion of the penetrating arteriole [25]. The current study augmented the postulated mechanism [12] for tES modulation by involving direct effects via neuron–vessel interactions [55]. Computationally immersed boundary method modeling for different vessel stiffness captured an immediate vascular peristaltic response, that may lead to a turbulent flow downstream in the stiffer vessels. Oscillatory tES perturbed vessel oscillations [71] and the effects on neurovascular coupling effects can be explored using joint imaging with NIRS-EEG [72]. Here, oscillatory tES hypothesized to acutely coordinate bottleneck penetrating arteriole peristalsis to satisfy metabolic needs, which is highlighted in Figure 2b. We posit in this paper that digital health integration with neurostimulation therapies for the therapeutic use of non-invasive brain stimulation might transform the complex neuroenergetic processes and the bulk flow of cerebrospinal fluid, enhancing favorable cognitive outcomes [42] in mild cognitive impairments to early dementia, such as diminished cognitive fatigue [73], attributed to improved extracellular clearance [74].

For oscillatory modal analysis, the linear model of the four physiologically detailed tES perturbation pathways was established using the Model Linearizer tool in the Simulink (MathWorks, Inc., USA) linear analysis package [12]. The damping ratio, natural frequency, and time constant of the poles were derived using the ‘damp’ function on the linear model system model of the four physiologically detailed tES perturbation pathways following the application of the Model Linearizer tool. Pathway 4 (see Figure 1a), from the tES perturbation effecting the voltage gated ion channels of smooth muscle cells in parts of the brain [75] to the change in the blood vessel circumference [12], i.e., the transfer function (TF4) in the Laplace domain, is

TF4 = (s + 2.962)/(s^6^ + 9.594 × 10^6^ s^5^ + 3.266 × 10^8^ s^4^ + 3.905 × 10^9^ s^3^ + 2.928 × 10^10^ s^2^ + 6.932 × 10^10^ s + 1.526 × 10^10^)

The damping ratio, natural frequency, and time constant of the poles for TF4 are
PoleDampingFrequency (rad/seconds)Time Constant (seconds)(−0.245 + 0 j)10.2454.09(−3.3 + 0 j)13.30.303(−4.9 + 8.44 j)0.5029.760.204(−4.9 − 8.44 j)0.5029.760.204(−20.7 + 0 j)120.70.0483(−9,590,000 + 0 j)19,590,0001.04 × 10^−7^

Note that for Pathway 4 for the tES perturbation effects, the natural frequency below 0.2 Hz is 0.04 Hz. Then, if we have the nested Pathway 3 (see Figure 1a) from the tES perturbation effecting the perivascular potassium concentration to the change in the blood vessel circumference [12], the transfer function (TF3) in the Laplace domain is

TF3 = (s^2^ + 2.371 × 10^7^ s + 7.023 × 10^7^)/(s^8^ + 9.624 × 10^6^ s^7^ + 2.857 × 10^11^ s^6^ + 1 × 10^13^ s^5^ + 1.259 × 10^14^ s^4^ + 9.87 × 10^14^ s^3^ + 2.932 × 10^15^ s^2^ + 2.515 × 10^15^ s + 4.538 × 10^14^)

The damping ratio, natural frequency, and time constant of the poles for TF3 are
PoleDampingFrequency (rad/seconds)Time Constant (seconds)(−0.245 + 0 j)10.2454.09(−1 + 0 j)111(−3.3 + 0 j)13.30.303(−4.9 + 8.44 j)0.5029.760.204(−4.9 − 8.44j)0.5029.760.204(−20.7+0j)120.70.0483(−29700 + 0 j)129,7003.36 × 10^−5^(−9,590,000 + 0 j)19,590,0001.04 × 10^−7^

Note that for Pathway 3 for the tES perturbation effects, the natural frequencies below 0.2 Hz are 0.04 Hz and 0.16 Hz. Then, if we have the nested Pathway 2 (see Figure 1a) from the tES perturbation effecting the astrocytic membrane potential to the change in the blood vessel circumference [12], the transfer function (TF2) in the Laplace domain is

TF2 = (s^3^ + 2.371 × 10^7^ s^2^ + 1.172 × 10^9^ s + 3.262 × 10^9^)/(s^10^ + 9.624 × 10^6^ s^9^ + 2.858 × 10^11^ s^8^ + 1.487 × 10^13^ s^7^ + 3.048 × 10^14^ s^6^ + 3.429 × 10^15^ s^5^ + 2.349 × 10^16^ s^4^ + 8.176 × 10^16^ s^3^ + 1.303 × 10^17^ s^2^ + 8.231 × 10^16^ s + 1.345 × 10^16^)

The damping ratio, natural frequency, and time constant of the poles for TF2 are
PoleDampingFrequency (rad/seconds)Time Constant (seconds)(−0.245 + 0 j)10.2454.09(−1 + 0 j)111(−1.97 + 0 j)11.970.509(−3.3 + 0 j)13.30.303(−4.9 + 8.44 j)0.5029.760.204(−4.9−8.44 j)0.5029.760.204(−15.1 + 0 j)115.10.0663(−20.7 + 0 j)120.70.0483(−29700 + 0 j)129,7003.36 × 10^−5^(−9,590,000 + 0 j)19,590,0001.04 × 10^−7^

Note that for the Pathway 2 for the tES perturbation effects, the natural frequencies below 0.2 Hz are 0.04 Hz and 0.16 Hz. Then, if we have the nested Pathway 1 (see Figure 1a) from the tES perturbation effecting the synaptic potassium to the change in the blood vessel circumference [12], the transfer function (TF1) in the Laplace domain is

TF1 = (s^3^ + 2.371 × 10^7^ s^2^ + 1.172 × 10^9^ s + 3.262 × 10^9^)/(s^11^ + 9.624 × 10^6^ s^10^ + 2.858 × 10^11^ s^9^ + 1.499 × 10^13^ s^8^ + 3.108 × 10^14^ s^7^ + 3.551 × 10^15^ s^6^ + 2.486 × 10^16^ s^5^ + 9.116 × 10^16^ s^4^ + 1.63 × 10^17^ s^3^ + 1.344 × 10^17^ s^2^ + 4.638 × 10^16^ s + 5.382 × 10^15^)

The damping ratio, natural frequency, and time constant of the poles for TF1 are
PoleDampingFrequency (rad/seconds)Time Constant (seconds)(−0.245 + 0 j)10.2454.09(−0.4 + 0 j)10.42.5(−1 + 0 j)111(−1.97 + 0 j)11.970.509(−3.3 + 0 j)13.30.303(−4.9 + 8.44 j)0.5029.760.204(−4.9−8.44 j)0.5029.760.204(−15.1 + 0 j)115.10.0663(−20.7 + 0 j)120.70.0483(−29,700 + 0 j)1297003.36 × 10^−5^(−9,590,000 + 0 j)19,590,0001.04 × 10^−7^

Note that for the Pathway 1 for the tES perturbation effects, the natural frequencies below 0.2 Hz are 0.04 Hz, 0.06 Hz, and 0.16 Hz. In order to test the modal analysis functions, including ‘modalfrf’, ‘modalfit’, and ‘modalsd’, using simulated data from the non-linear models of four physiologically detailed tES perturbation pathways [12], the input of transcranial electrical stimulation (tES) and the corresponding vessel responses for the proposed pathways were investigated. We subjected the four compartmental pathways to tOCS (combined tDCS and tACS) perturbations with varying frequencies (ranging from 0.1 Hz to 10 Hz) and direct current (DC) offsets (ranging from 0 to 2 mA). Subsequently, we conducted a sensitivity analysis to assess changes in the blood vessel circumference. 

We considered three model input waveforms as follows.

(1) $$tESinputs:\left\{\begin{array}{l} c\;for\;tDCS\\ a\;\sin\left(2\pi ft\right)\;for\;tACS\\ a\;\sin\left(2\pi ft\right)+c\;for\;tOCS \end{array}\right.$$

**Figure 3:**
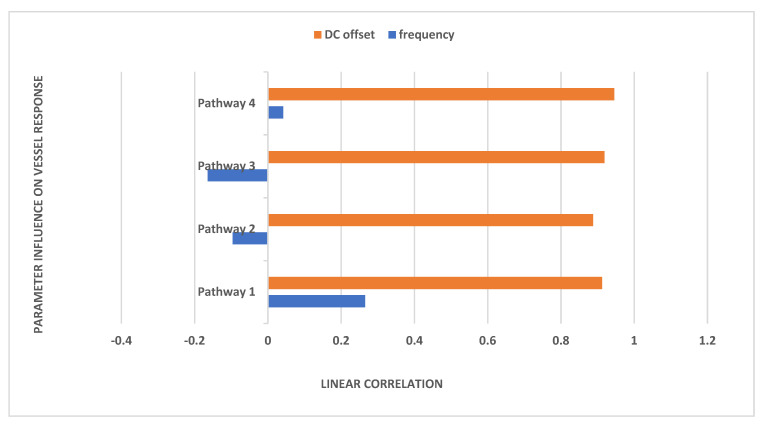
Effect of frequency (tDCS has frequency 0) and DC offset of tOCS on vessel circumference based sensitivity analysis for the proposed pathways.

In Equation (1), we considered a sinusoidal amplitude (*a*) of 1 mA, a sinusoidal frequency (*f*) ranging from 0.1 to 10 Hz, and a DC offset (*c*) ranging from 0 to 2 times the amplitude for conducting a sensitivity analysis on the physiologically detailed mathematical model of the neurovascular unit. This analysis, facilitated by the Sensitivity Analyzer tool in MATLAB Simulink (MathWorks, Inc., USA), enabled the exploration of the tES design space, identifying the most influential model parameters. Figure 3 illustrates the impact of frequency and DC offset on the vessel response with tOCS @ 0.1 Hz performing the best across all the pathways. Then, Pathway 4 was most responsive to tACS and tOCS @ 1 Hz. Pathways 1 and 4 were entrained with a positive correlation while Pathways 2 and 3 were entrained with a negative correlation for the tACS part of the tOCS—see Figure 3.

**Figure 4:**
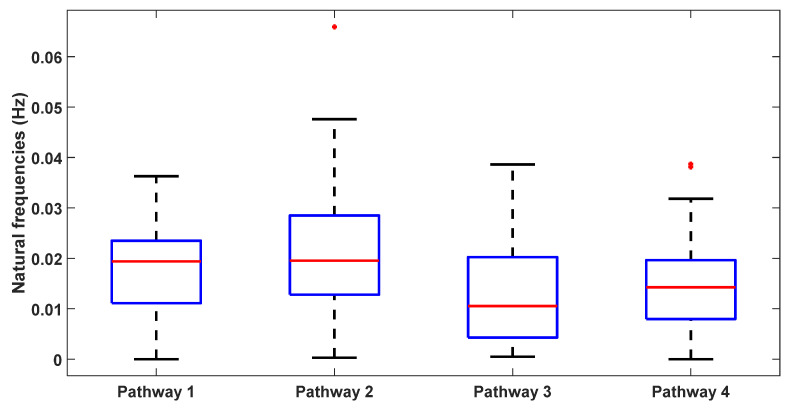
Boxplot illustrates the distribution of natural frequencies resulting from modal analysis (with different seed values) for the four transcranial electrical stimulation (tES) perturbation model pathways that are <0.1 Hz. Each box represents the interquartile range, with the central mark denoting the median. The bottom and top edges of the box indicate the 25th and 75th percentiles, respectively. Whiskers extend to the most extreme data points that are not considered outliers, while outliers are individually marked with a red ‘+’ symbol.

**Figure 5:**
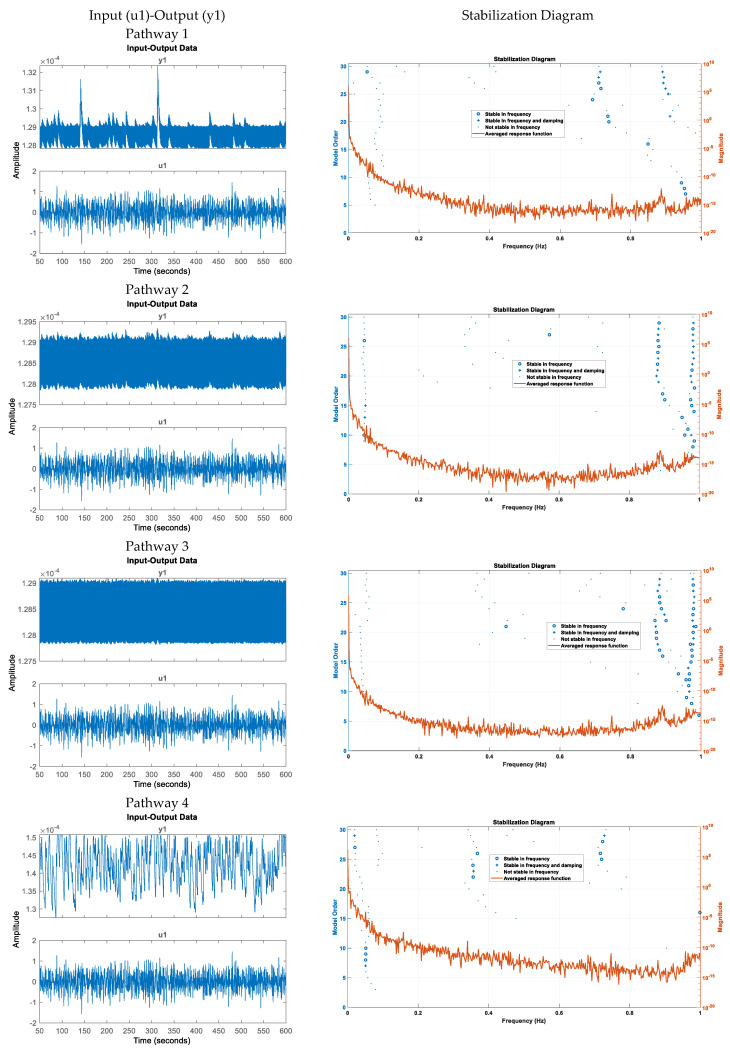
Stabilization diagram obtained for the first case (model input: bandpass-filtered white noise, default seed value) for the four pathways. Pathway 4 has robust stable modes < 0.1 Hz while Pathways 1 to 3 have also robust stable modes > 0.8 Hz across model order. We can find stable modes ~0.05 Hz for all the pathways of the neurovascular unit since Pathways 1 to 3 have nested Pathway 4—see Figure 1a.

Table 1 presents the natural frequencies derived through the modal analysis of the non-linear models of four physiologically detailed tES perturbation pathways under a Bandpass-filtered White Noise Input and Figure 4 illustrates a boxplot representation of these frequencies. Additionally, Figure 5 displays the stabilization diagram obtained for the first case, where the model input was bandpass-filtered white noise with the default seed value for all the four pathways. Table 1 lists the system parameters associated with a linearized model for the four pathways—Pathway 1 involves tES perturbing the vessel response through the synaptic potassium pathway, Pathway 2 through the astrocytic pathway, Pathway 3 through the perivascular potassium pathway, and Pathway 4 through the smooth muscle cell pathway. Figure 5 showed the natural frequencies of the non-linear models of four physiologically detailed tES perturbation pathways where we can find stable modes ~0.05 Hz for all the pathways that were compatible with the linearized model analysis (0.04 Hz, 0.06 Hz).

## 4. Discussion

Our results demonstrate the potential of tES in manipulating perivascular nerve activity, thereby modulating blood flow dynamics in cerebral arterioles. Specifically, our findings from Figure 2b support the hypothesis that tES can induce initial vasoconstriction (“initial dip”) in pial arteries, which facilitates downstream blood flow enhancement. This effect is likely complemented by a delayed vasodilation response due to metabolic demands in upstream penetrating arterioles, a phenomenon that may be particularly beneficial in managing vascular cognitive impairments. Moreover, our study incorporates the FitzHugh–Nagumo model to simulate the electrophysiological behavior of perivascular nerves along penetrating arterioles, highlighting how tES can influence the vascular diameter through synaptic-like interactions at neural–aSMC junctions. These interactions may be critical in conditions characterized by increased vascular stiffness, such as small vessel disease (SVD) seen in diabetes. Elevated glucose levels in diabetes contribute to structural changes in vessel proteins, which can affect overall neurovascular coupling—a key factor in dementia progression. Our computational modeling also suggests that variations in the modulus of the elasticity of vessel walls can significantly affect hemodynamic responses, such as the flow velocity and turbulence, potentially impacting endothelial and glycocalyx functions. Such insights are crucial for understanding the mechanistic underpinnings of tES in therapeutic contexts, particularly its role in enhancing neurovascular coupling and cognitive function. Indeed, a hallmark of dementia syndromes is an energy deficit in neurons, leading to synaptic loss and resulting in cognitive decline and behavioral changes [76]; therefore, an energy boost is necessary [77], e.g., via facilitating neurovascular [78] and neurometabolic [79] coupling rather than solely excitatory tDCS that may increase the energy deficit leading to synaptic loss. Also, Gamma entrainment may positively impact excitation/inhibition imbalances by preventing neurochemical changes linked to Aβ, such as hyperexcitability, and triggering neuroprotective mechanisms [80]. However, we should be aware of the energy cost to avoid a deficit (and cell death) due to Gamma entrainment since fast-spiking interneurons, with their membrane potentials around 10 to 15 mV, which is more depolarized than those of pyramidal cells, may incur higher energy costs due to the need for chloride extrusion [81]. Additionally, the dynamic balance between excitatory and inhibitory synaptic inputs can challenge ion homeostasis. For example, an increase in inhibition typically coincides with an increase in excitation, maintaining excitability within a functional range. However, in high membrane conductance states, this balance leads to heightened ion fluxes for excitatory transmission, which can significantly increase the energy demands on both inhibitory interneurons and excitatory pyramidal cells during periods of intense synaptic activity due to Gamma entrainment. 

There are various neurostimulation approaches to facilitate CBF: Animal studies have revealed the presence of spontaneous “hypoxic pockets” in the brains of awake, active mice, linked to interruptions in the local capillary flow [82]. Moreover, it was found that exercise reduced these hypoxic areas by 52% compared to when at rest. This research offers new understanding of the oxygen distribution in the brain and introduces interventions to target oxygen levels in physiological functions and cerebrovascular disorders. Here, peripheral neuromuscular electrical stimulation (NMES) can have beneficial cerebral hemodynamic effects [83] that can be adjuvant to exercise interventions where peripheral NMES effects can be partly due to the somato-autonomic reflex. We postulate, for multi-level non-invasive electrical stimulation interventions, that the orthosympathetic and the LC-NA system effects need to be delineated since they can have opposite effects on CBF. Also, different forms of tES have varying current profiles for tES—transcranial direct current stimulation (tDCS) uses a monophasic, non-oscillating baseline, while transcranial alternating current stimulation (tACS) involves rhythmically reversing the electrical current. Other methods include transcranial oscillating current stimulation (tOCS), using a direct component to guide oscillations, and transcranial random noise stimulation (tRNS), injecting an alternating current with bounded stochasticity [84,85]. Neurovascular modulation occurs in various stimulation protocols, with mechanisms not fully understood that are postulated to be partly driven by somato-autonomic [86] and the LC-NA [12] effects. As tES affects blood vessels through neuronal or non-neuronal cells, a deeper understanding of signaling pathways is crucial [12]. Here, tACS, unique in manipulating cell-class specific [87] intrinsic oscillations possibly through ephaptic coupling [88], may hold promise for addressing vascular dementia [89,90] and related microvascular dysfunction. In the systematic review by Machado and colleagues [91] and meta-analysis of 22 studies involving 393 participants, the effects of transcranial Direct Current Stimulation (tDCS) on exercise performance were examined. Weak evidence suggested a significant positive effect of anodal tDCS (a-tDCS) over the motor cortex (M1) on time to exhaustion (TTE) in cycling, but the results were influenced by a single study. No significant effects were found for cathodal tDCS (c-tDCS) on TTE. For isometric muscle strength, no significant effects were observed for a-tDCS applied before or during exercise. Mixed results were reported for isokinetic muscle strength. A quantitative synthesis indicated a significant improvement in cycling performance with a-tDCS over M1, but caution was advised due to the influence of a single study. Commercial tDCS devices for exercise performance were not addressed, raising safety and efficacy concerns. Methodological aspects, including individual variability and optimal tDCS parameters, need further exploration in future research. Here, tES has a role in improving exercise performance when individually customized for priming the NVU for optimal hemodynamic responses in a closed-loop manner [2] while addressing detrimental cerebral effects of the metaboreflex [92,93] using peripheral NMES. Human in the loop approach [2] via ephaptic coupling [88] may leverage the synaptic transmission between the neural axons and the smooth muscle cells in arterioles [55] thereby modulating the neurovascular pathway [12].

Limitations: In our mathematical model, we did not present a spatio-temporal tES approach to facilitate long-wavelength traveling waves of vasomotion, which is a major limitation and needs to be addressed in the future. Brain arterioles, oscillating at approximately 0.1 Hz, are active multicellular complexes [94]. In awake mice, these vaso-oscillations in penetrating arterioles significantly impact neocortex perfusion, with resting-state activity modulating blood flow more than stimulus-induced activity. The weak relationship between arteriole diameter changes and perfusion suggests that the capillary bed predominantly controls brain vasculature resistance. The phase of these oscillations evolves slowly along arterioles, creating cortical areas with uniform phases which may be measured using whole-head multi-distance fNIRS with spatio-temporal oscillatory modal analysis [95] and spatiotemporal dynamic mode decomposition [96]—see the Supplementary Materials for the theoretical development [48,97] using coupled complex Ginzburg-Landau equations [98,99,100]. Here, phase-gradient supports bidirectional traveling waves, indicating that penetrating arteriole waves can mix, but not directional transport of interstitial fluids, which needs further investigation in future works.

## 5. Conclusions

This study provides important insights into the vascular mechanisms activated by tES and their potential therapeutic implications for neurovascular and cognitive dysfunctions. The ability of tES to induce coordinated peristaltic motion in penetrating arterioles could be a significant factor in enhancing cerebral blood flow and, consequently, cognitive functions in patients with vascular impairments. Then, the integration of tES with digital health technologies at the point-of-care could revolutionize the management of cognitive impairments in early dementia stages, potentially reducing cognitive fatigue and enhancing extracellular clearance through improved neurovascular coupling. Future research should focus on refining these neurostimulation strategies, optimizing their parameters, and validating their effectiveness in clinical settings to fully harness their therapeutic potential. These findings open new avenues for the use of neuromodulation in treating vascular and neurodegenerative diseases, emphasizing the need for further investigation into the optimal integration of these technologies into clinical practice.

## Figures and Tables

**Table 1:** Natural frequencies (Hz) obtained from the modal analysis (see also Figure 4) of the four physiologically detailed tES perturbation pathways for ten different seed values for the Bandpass-filtered White Noise Input (1* presents the results from the default seed value).

Pathway/Bandpass-Filtered White Noise Input	P1 (K1 = 0.000001) Hz	P2 (K2 = 0.00000000001) Hz	P3 (K3 = 0.001) Hz	P4 (K4 = 0.000000000001) Hz
1*	0.0193	0.0247	0.0052, 0.0157, 0.0265	0.0069, 0.0201
2	0.0189, 0.0201	0.0003, 0.0156, 0.0167	0.0025, 0.0231, 0.0235	0.0128, 0.0141
3	0.0153, 0.0195	0.0109, 0.0191, 0.0288, 0.0476	0.0027, 0.0081, 0.0196, 0.0236	0.0067, 0.0090, 0.0144
4	0.0115, 0.0245	-	0.0050	0.0020, 0.0168
5	0.0007, 0.0077, 0.0200, 0.0276	0.0131	0.0005, 0.0128, 0.0176,	0.0192, 0.0318
6	0.0179, 0.0363	0.0237, 0.0418	0.0055	0.0104, 0.0171
7	0.0107, 0.0131	0.0128, 0.0200, 0.0380, 0.0659	0.0036, 0.0386	0.0381
8	0.0225, 0.0298	0.0048, 0.0100	0.0011, 0.0209,	0.0043, 0.0223
9	0.0215	0.0038, 0.0285	0.0033, 0.0083, 0.0184	0.0099, 0.0164
10	0.0198, 0.0319, 0.0360	0.0189, 0.0229, 0.0242	0.0058, 0.0188	0.0387

## Data Availability

Data are contained within the article.

## Supplementary Materials

The following supporting information can be downloaded at: https://www.mdpi.com/article/10.3390/brainsci14060591/s1, Supplementary File S1.

## References

[1] Zheng X., Alsop D.C., Schlaug G. (2011). Effects of Transcranial Direct Current Stimulation (tDCS) on Human Regional Cerebral Blood Flow. NeuroImage.

[2] Arora Y., Dutta A. (2022). Human-in-the-Loop Optimization of Transcranial Electrical Stimulation at the Point of Care: A Computational Perspective. Brain Sci..

[3] Sara S.J., Bouret S. (2012). Orienting and Reorienting: The Locus Coeruleus Mediates Cognition through Arousal. Neuron.

[4] Witts E.C., Mathews M.A., Murray A.J. (2023). The Locus Coeruleus Directs Sensory-Motor Reflex Amplitude across Environmental Contexts. Curr. Biol. CB.

[5] Ogoh S., Tarumi T. (2019). Cerebral Blood Flow Regulation and Cognitive Function: A Role of Arterial Baroreflex Function. J. Physiol. Sci..

[6] Silvani A., Calandra-Buonaura G., Benarroch E.E., Dampney R.A.L., Cortelli P. (2015). Bidirectional Interactions between the Baroreceptor Reflex and Arousal: An Update. Sleep Med..

[7] Silva-Filho E., Albuquerque J., Bikson M., Pegado R., da Cruz Santos A., do Socorro Brasileiro-Santos M. (2021). Effects of Transcranial Direct Current Stimulation Associated with an Aerobic Exercise Bout on Blood Pressure and Autonomic Modulation of Hypertensive Patients: A Pilot Randomized Clinical Trial. Auton. Neurosci..

[8] Rodrigues B., Barboza C.A., Moura E.G., Ministro G., Ferreira-Melo S.E., Castaño J.B., Nunes W.M.S., Mostarda C., Coca A., Vianna L.C. (2022). Acute and Short-Term Autonomic and Hemodynamic Responses to Transcranial Direct Current Stimulation in Patients With Resistant Hypertension. Front. Cardiovasc. Med..

[9] Yu T.Y., Lee M. (2021). Autonomic Dysfunction, Diabetes and Metabolic Syndrome. J. Diabetes Investig..

[10] Pillai J.A., Bena J., Bekris L., Kodur N., Kasumov T., Leverenz J.B., Kashyap S.R., on behalf of the Alzheimer’s Disease Neuroimaging Initiative (2023). Metabolic Syndrome Biomarkers Relate to Rate of Cognitive Decline in MCI and Dementia Stages of Alzheimer’s Disease. Alzheimers Res. Ther..

[11] Giorgi F.S., Galgani A., Puglisi-Allegra S., Limanaqi F., Busceti C.L., Fornai F. (2020). Locus Coeruleus and Neurovascular Unit: From Its Role in Physiology to Its Potential Role in Alzheimer’s Disease Pathogenesis. J. Neurosci. Res..

[12] Arora Y., Walia P., Hayashibe M., Muthalib M., Chowdhury S.R., Perrey S., Dutta A. (2021). Grey-Box Modeling and Hypothesis Testing of Functional near-Infrared Spectroscopy-Based Cerebrovascular Reactivity to Anodal High-Definition tDCS in Healthy Humans. PLOS Comput. Biol..

[13] Modal Analysis—1st Edition. https://shop.elsevier.com/books/modal-analysis/fu/978-0-7506-5079-3.

[14] Modal Analysis for Damage Detection in Structures—Semantic Scholar. https://www.semanticscholar.org/paper/Modal-Analysis-for-Damage-Detection-in-Structures-Hearn-Testa/38a4691979855d965640f076820b6acc406feae9.

[15] Rogers G., Rogers G. (2000). Modal Analysis for Control. Power System Oscillations.

[16] Dutta A., Jacob A., Chowdhury S.R., Das A., Nitsche M.A. (2015). EEG-NIRS Based Assessment of Neurovascular Coupling during Anodal Transcranial Direct Current Stimulation—A Stroke Case Series. J. Med. Syst..

[17] Sood M., Besson P., Muthalib M., Jindal U., Perrey S., Dutta A., Hayashibe M. (2016). NIRS-EEG Joint Imaging during Transcranial Direct Current Stimulation: Online Parameter Estimation with an Autoregressive Model. J. Neurosci. Methods.

[18] Arora Y., Dutta A. (2023). Perspective: Disentangling the Effects of tES on Neurovascular Unit. Front. Neurol..

[19] Wu G.-R., Colenbier N., Van Den Bossche S., Clauw K., Johri A., Tandon M., Marinazzo D. (2021). rsHRF: A Toolbox for Resting-State HRF Estimation and Deconvolution. NeuroImage.

[20] Shin D.W., Fan J., Luu E., Khalid W., Xia Y., Khadka N., Bikson M., Fu B.M. (2020). In Vivo Modulation of the Blood-Brain Barrier Permeability by Transcranial Direct Current Stimulation (tDCS). Ann. Biomed. Eng..

[21] Hertz L., Lovatt D., Goldman S.A., Nedergaard M. (2010). Adrenoceptors in Brain: Cellular Gene Expression and Effects on Astrocytic Metabolism and [Ca2+]i. Neurochem. Int..

[22] Moshkforoush A., Ashenagar B., Harraz O.F., Dabertrand F., Longden T.A., Nelson M.T., Tsoukias N.M. (2020). The Capillary Kir Channel as Sensor and Amplifier of Neuronal Signals: Modeling Insights on K+-Mediated Neurovascular Communication. Proc. Natl. Acad. Sci..

[23] Reitman M.E., Tse V., Mi X., Willoughby D.D., Peinado A., Aivazidis A., Myagmar B.-E., Simpson P.C., Bayraktar O.A., Yu G. (2023). Norepinephrine Links Astrocytic Activity to Regulation of Cortical State. Nat. Neurosci..

[24] Vanneste S., Mohan A., Yoo H.B., Huang Y., Luckey A.M., McLeod S.L., Tabet M.N., Souza R.R., McIntyre C.K., Chapman S. (2020). The Peripheral Effect of Direct Current Stimulation on Brain Circuits Involving Memory. Sci. Adv..

[25] Dutta A., Tomita M., Lahiri U., Das A. NeuroMind: Eye Tracking and Portable Neuroimaging to Monitor Multidomain Lifestyle Intervention for Healthy Ageing, In Proceedings of the Dementia Research at UCLan and Beyond: An Event for Scientists and the Public, Preston, UK, 21 July 2023.

[26] Sheng Y., Zhu L. (2018). The Crosstalk between Autonomic Nervous System and Blood Vessels. Int. J. Physiol. Pathophysiol. Pharmacol..

[27] Kuo H., Paulus W., Batsikadze G., Jamil A., Kuo M., Nitsche M.A. (2017). Acute and Chronic Effects of Noradrenergic Enhancement on Transcranial Direct Current Stimulation-induced Neuroplasticity in Humans. J. Physiol..

[28] Khadka N., Bikson M. (2020). Neurocapillary-Modulation. Neuromodul. J. Int. Neuromodul. Soc..

[29] Bleys R.L.A.W., Cowen T., Groen G.J., Hillen B., Ibrahim N.B.N. (1996). Perivascular Nerves of the Human Basal Cerebral Arteries: I. Topographical Distribution. J. Cereb. Blood Flow Metab..

[30] Grizzanti J., Moritz W.R., Pait M.C., Stanley M., Kaye S.D., Carroll C.M., Constantino N.J., Deitelzweig L.J., Snipes J.A., Kellar D. (2023). KATP Channels Are Necessary for Glucose-Dependent Increases in Amyloid-β and Alzheimer’s Disease–Related Pathology. JCI Insight.

[31] Bekar L.K., Wei H.S., Nedergaard M. (2012). The Locus Coeruleus-Norepinephrine Network Optimizes Coupling of Cerebral Blood Volume with Oxygen Demand. J. Cereb. Blood Flow Metab..

[32] Korte N., James G., You H., Hirunpattarasilp C., Christie I., Sethi H., Attwell D. (2023). Noradrenaline Released from Locus Coeruleus Axons Contracts Cerebral Capillary Pericytes via A2 Adrenergic Receptors. J. Cereb. Blood Flow Metab. Off. J. Int. Soc. Cereb. Blood Flow Metab..

[33] Zhang L., Fang Z., Chen W. (2012). Quick and Effective Hyperpolarization of the Membrane Potential in Intact Smooth Muscle Cells of Blood Vessels by Synchronization Modulation Electric Field. J. Bioenerg. Biomembr..

[34] Koep J.L., Taylor C.E., Coombes J.S., Bond B., Ainslie P.N., Bailey T.G. (2022). Autonomic Control of Cerebral Blood Flow: Fundamental Comparisons between Peripheral and Cerebrovascular Circulations in Humans. J. Physiol..

[35] Claassen J.A.H.R., Thijssen D.H.J., Panerai R.B., Faraci F.M. (2021). Regulation of Cerebral Blood Flow in Humans: Physiology and Clinical Implications of Autoregulation. Physiol. Rev..

[36] LONGDEN T.A., NELSON M.T. (2015). Vascular Inward Rectifier K+ Channels as External K+ Sensors in the Control of Cerebral Blood Flow. Microcirc. N. Y. N 1994.

[37] Zhao F., Tomita M., Dutta A. (2023). Operational Modal Analysis of Near-Infrared Spectroscopy Measure of 2-Month Exercise Intervention Effects in Sedentary Older Adults with Diabetes and Cognitive Impairment. Brain Sci..

[38] Mauri P., Miniussi C., Balconi M., Brignani D. (2015). Bursts of Transcranial Electrical Stimulation Increase Arousal in a Continuous Performance Test. Neuropsychologia.

[39] Piccirillo G., Ottaviani C., Fiorucci C., Petrocchi N., Moscucci F., Di Iorio C., Mastropietri F., Parrotta I., Pascucci M., Magrì D. (2016). Transcranial Direct Current Stimulation Improves the QT Variability Index and Autonomic Cardiac Control in Healthy Subjects Older than 60 Years. Clin. Interv. Aging.

[40] Sherwood M.S., Madaris A.T., Mullenger C.R., McKinley R.A. (2018). Repetitive Transcranial Electrical Stimulation Induces Quantified Changes in Resting Cerebral Perfusion Measured from Arterial Spin Labeling. Neural Plast..

[41] Dutta A. (2015). Bidirectional Interactions between Neuronal and Hemodynamic Responses to Transcranial Direct Current Stimulation (tDCS): Challenges for Brain-State Dependent tDCS. Front. Syst. Neurosci..

[42] Dutta A. (2021). Simultaneous Functional Near-Infrared Spectroscopy (fNIRS) and Electroencephalogram (EEG) to Elucidate Neurovascular Modulation by Transcranial Electrical Stimulation (tES). Brain Stimulat..

[43] Steenland K., Karnes C., Seals R., Carnevale C., Hermida A., Levey A. (2012). Late-Life Depression as a Risk Factor for Mild Cognitive Impairment or Alzheimer’s Disease in 30 US Alzheimer’s Disease Centers. J. Alzheimers Dis. JAD.

[44] Najjar S., Pearlman D.M., Devinsky O., Najjar A., Zagzag D. (2013). Neurovascular Unit Dysfunction with Blood-Brain Barrier Hyperpermeability Contributes to Major Depressive Disorder: A Review of Clinical and Experimental Evidence. J. Neuroinflammation.

[45] Knotkova H., Nitsche M.A., Bikson M., Woods A.J. (2019). Practical Guide to Transcranial Direct Current Stimulation: Principles, Procedures and Applications.

[46] Bergmann T.O., Groppa S., Seeger M., Mölle M., Marshall L., Siebner H.R. (2009). Acute Changes in Motor Cortical Excitability during Slow Oscillatory and Constant Anodal Transcranial Direct Current Stimulation. J. Neurophysiol..

[47] Vieira P.G., Krause M.R., Pack C.C. (2020). tACS Entrains Neural Activity While Somatosensory Input Is Blocked. PLoS Biol..

[48] Taira K., Brunton S.L., Dawson S.T.M., Rowley C.W., Colonius T., McKeon B.J., Schmidt O.T., Gordeyev S., Theofilis V., Ukeiley L.S. (2017). Modal Analysis of Fluid Flows: An Overview. AIAA J..

[49] Gruszecki M., Nuckowska M.K., Szarmach A., Radkowski M., Szalewska D., Waskow M., Szurowska E., Frydrychowski A.F., Demkow U., Winklewski P.J. (2018). Oscillations of Subarachnoid Space Width as a Potential Marker of Cerebrospinal Fluid Pulsatility. Adv. Exp. Med. Biol..

[50] Svinkunaite L., Horschig J.M., Floor-Westerdijk M.J. (2021). Employing Cardiac and Respiratory Features Extracted from fNIRS Signals for Mental Workload Classification. Proceedings of the Biophotonics in Exercise Science, Sports Medicine, Health Monitoring Technologies, and Wearables II.

[51] Mestre H., Tithof J., Du T., Song W., Peng W., Sweeney A.M., Olveda G., Thomas J.H., Nedergaard M., Kelley D.H. (2018). Flow of Cerebrospinal Fluid Is Driven by Arterial Pulsations and Is Reduced in Hypertension. Nat. Commun..

[52] Vijayakrishnan Nair V., Kish B.R., Inglis B., Yang H.-C., Wright A.M., Wu Y.-C., Zhou X., Schwichtenberg A.J., Tong Y. (2022). Human CSF Movement Influenced by Vascular Low Frequency Oscillations and Respiration. Front. Physiol..

[53] Murdock M.H., Yang C.-Y., Sun N., Pao P.-C., Blanco-Duque C., Kahn M.C., Kim T., Lavoie N.S., Victor M.B., Islam M.R. (2024). Multisensory Gamma Stimulation Promotes Glymphatic Clearance of Amyloid. Nature.

[54] Jiang-Xie L.-F., Drieu A., Bhasiin K., Quintero D., Smirnov I., Kipnis J. (2024). Neuronal Dynamics Direct Cerebrospinal Fluid Perfusion and Brain Clearance. Nature.

[55] Zhang D., Ruan J., Peng S., Li J., Hu X., Zhang Y., Zhang T., Ge Y., Zhu Z., Xiao X. (2024). Synaptic-like Transmission between Neural Axons and Arteriolar Smooth Muscle Cells Drives Cerebral Neurovascular Coupling. Nat. Neurosci..

[56] Faghih M.M., Sharp M.K. (2018). Is Bulk Flow Plausible in Perivascular, Paravascular and Paravenous Channels?. Fluids Barriers CNS.

[57] Silver I.A., Erecińska M. (1994). Extracellular Glucose Concentration in Mammalian Brain: Continuous Monitoring of Changes during Increased Neuronal Activity and upon Limitation in Oxygen Supply in Normo-, Hypo-, and Hyperglycemic Animals. J. Neurosci. Off. J. Soc. Neurosci..

[58] Pinti P., Tachtsidis I., Hamilton A., Hirsch J., Aichelburg C., Gilbert S., Burgess P.W. (2020). The Present and Future Use of Functional Near-Infrared Spectroscopy (fNIRS) for Cognitive Neuroscience. Ann. N. Y. Acad. Sci..

[59] Kim Y.-K., Nam K.I., Song J. (2018). The Glymphatic System in Diabetes-Induced Dementia. Front. Neurol..

[60] Xie L., Kang H., Xu Q., Chen M.J., Liao Y., Thiyagarajan M., O’Donnell J., Christensen D.J., Nicholson C., Iliff J.J. (2013). Sleep Drives Metabolite Clearance from the Adult Brain. Science.

[61] Mukli P., Csipo T., Lipecz A., Stylianou O., Racz F.S., Owens C.D., Perry J.W., Tarantini S., Sorond F.A., Kellawan J.M. (2021). Sleep Deprivation Alters Task-Related Changes in Functional Connectivity of the Frontal Cortex: A near-Infrared Spectroscopy Study. Brain Behav..

[62] Lanza G., DelRosso L.M., Ferri R. (2022). Sleep and Homeostatic Control of Plasticity. Handb. Clin. Neurol..

[63] Nitsche M.A., Fricke K., Henschke U., Schlitterlau A., Liebetanz D., Lang N., Henning S., Tergau F., Paulus W. (2003). Pharmacological Modulation of Cortical Excitability Shifts Induced by Transcranial Direct Current Stimulation in Humans. J. Physiol..

[64] Esmaeilpour Z., Kronberg G., Reato D., Parra L.C., Bikson M. (2020). Temporal Interference Stimulation Targets Deep Brain Regions by Modulating Neural Oscillations. Brain Stimulat..

[65] Cao J., Doiron B., Goswami C., Grover P. (2020). The Mechanics of Temporal Interference Stimulation. BioRxiv.

[66] Nishimura N., Schaffer C.B., Friedman B., Lyden P.D., Kleinfeld D. (2007). Penetrating Arterioles Are a Bottleneck in the Perfusion of Neocortex. Proc. Natl. Acad. Sci. USA.

[67] Cheung M.C. (2018). Hemodynamics Due to Calf Muscle Activity–Biophysical Modeling and Experiments Using Frequency Domain near Infrared Spectroscopy in Healthy Humans. Master’s Thesis.

[68] Battista N.A., Strickland W.C., Barrett A., Miller L.A. (2018). IB2d Reloaded: A More Powerful Python and MATLAB Implementation of the Immersed Boundary Method. Math. Methods Appl. Sci..

[69] Liebetanz D., Nitsche M.A., Tergau F., Paulus W. (2002). Pharmacological Approach to the Mechanisms of Transcranial DC-Stimulation-Induced after-Effects of Human Motor Cortex Excitability. Brain J. Neurol..

[70] Hariharan A., Robertson C.D., Garcia D.C.G., Longden T.A. (2022). Brain Capillary Pericytes Are Metabolic Sentinels That Control Blood Flow through a KATP Channel-Dependent Energy Switch. Cell Rep..

[71] Arora Y., Chowdhury S.R., Dutta A. (2021). Physiological Neurovascular Modeling of Cerebrovascular Effects of Transcranial Electrical Current Stimulation. Brain Stimul. Basic Transl. Clin. Res. Neuromodul..

[72] (2015). Development of Point of Care Testing Device for Neurovascular Coupling From Simultaneous Recording of EEG and NIRS During Anodal Transcranial Direct Current Stimulation. IEEE J. Transl. Eng. Health Med..

[73] Wiehler A., Branzoli F., Adanyeguh I., Mochel F., Pessiglione M. (2022). A Neuro-Metabolic Account of Why Daylong Cognitive Work Alters the Control of Economic Decisions. Curr. Biol..

[74] Tarasoff-Conway J.M., Carare R.O., Osorio R.S., Glodzik L., Butler T., Fieremans E., Axel L., Rusinek H., Nicholson C., Zlokovic B.V. (2015). Clearance Systems in the Brain—Implications for Alzheimer Disease. Nat. Rev. Neurol..

[75] Tykocki N.R., Boerman E.M., Jackson W.F. (2017). Smooth Muscle Ion Channels and Regulation of Vascular Tone in Resistance Arteries and Arterioles. Compr. Physiol..

[76] Yassine H.N., Solomon V., Thakral A., Sheikh-Bahaei N., Chui H.C., Braskie M.N., Schneider L.S., Talbot K. (2022). Brain Energy Failure in Dementia Syndromes: Opportunities and Challenges for Glucagon-like Peptide-1 Receptor Agonists. Alzheimers Dement..

[77] Mallucci G. (2024). Dementia Therapy: Time for an Energy Boost. Brain.

[78] Mukli P., Pinto C.B., Owens C.D., Csipo T., Lipecz A., Szarvas Z., Peterfi A., Langley A.C.d.C.P., Hoffmeister J., Racz F.S. (2024). Impaired Neurovascular Coupling and Increased Functional Connectivity in the Frontal Cortex Predict Age-Related Cognitive Dysfunction. Adv. Sci..

[79] Shahdadian S., Wang X., Liu H. (2024). Directed Physiological Networks in the Human Prefrontal Cortex at Rest and Post Transcranial Photobiomodulation. Sci. Rep..

[80] Manippa V., Palmisano A., Filardi M., Vilella D., Nitsche M.A., Rivolta D., Logroscino G. (2022). An Update on the Use of Gamma (Multi)Sensory Stimulation for Alzheimer’s Disease Treatment. Front. Aging Neurosci..

[81] Kann O., Papageorgiou I.E., Draguhn A. (2014). Highly Energized Inhibitory Interneurons Are a Central Element for Information Processing in Cortical Networks. J. Cereb. Blood Flow Metab..

[82] Beinlich F.R.M., Asiminas A., Untiet V., Bojarowska Z., Plá V., Sigurdsson B., Timmel V., Gehrig L., Graber M.H., Hirase H. (2024). Oxygen Imaging of Hypoxic Pockets in the Mouse Cerebral Cortex. Science.

[83] Dutta A., Zhao F., Cheung M., Das A., Tomita M., Chatterjee K. Cerebral and Muscle Near-Infrared Spectroscopy during Lower-Limb Muscle Activity – Volitional and Neuromuscular Electrical Stimulation. Proceedings of the 2021 43rd Annual International Conference of the IEEE Engineering in Medicine & Biology Society (EMBC).

[84] Guleyupoglu B., Schestatsky P., Edwards D., Fregni F., Bikson M. (2013). Classification of Methods in Transcranial Electrical Stimulation (tES) and Evolving Strategy from Historical Approaches to Contemporary Innovations. J. Neurosci. Methods.

[85] Paulus W. (2011). Transcranial Electrical Stimulation (tES*—*tDCS; tRNS, tACS) Methods. Neuropsychol. Rehabil..

[86] Rezaee Z., Dutta A. Transcranial Direct Current Stimulation of the Leg Motor Area*—*Is It Partly Somatosensory?. Proceedings of the 2018 40th Annual International Conference of the IEEE Engineering in Medicine and Biology Society (EMBC).

[87] Lee S.Y., Kozalakis K., Baftizadeh F., Campagnola L., Jarsky T., Koch C., Anastassiou C.A. (2024). Cell-Class-Specific Electric Field Entrainment of Neural Activity. Neuron.

[88] Dutta A., Dutta A. Using Electromagnetic Reciprocity and Magnetic Resonance Current Density Imaging to Fit Multi-Electrode Montage for Non-Invasive Brain Stimulation. Proceedings of the 2013 6th International IEEE/EMBS Conference on Neural Engineering (NER).

[89] Antal A., Paulus W. (2013). Transcranial Alternating Current Stimulation (tACS). Front. Hum. Neurosci..

[90] Helfrich R.F., Schneider T.R., Rach S., Trautmann-Lengsfeld S.A., Engel A.K., Herrmann C.S. (2014). Entrainment of Brain Oscillations by Transcranial Alternating Current Stimulation. Curr. Biol..

[91] Machado D.G.d.S., Unal G., Andrade S.M., Moreira A., Altimari L.R., Brunoni A.R., Perrey S., Mauger A.R., Bikson M., Okano A.H. (2019). Effect of Transcranial Direct Current Stimulation on Exercise Performance: A Systematic Review and Meta-Analysis. Brain Stimulat..

[92] Roberto S., Milia R., Doneddu A., Pinna V., Palazzolo G., Serra S., Orrù A., Hosseini Kakhak S.A., Ghiani G., Mulliri G. (2019). Hemodynamic Abnormalities during Muscle Metaboreflex Activation in Patients with Type 2 Diabetes Mellitus. J. Appl. Physiol. Bethesda Md 1985.

[93] Doneddu A., Roberto S., Pinna V., Magnani S., Ghiani G., Sainas G., Mulliri G., Serra S., Kakhak S.A.H., Milia R. (2020). Effect of Combined Mental Task and Metaboreflex Activation on Hemodynamics and Cerebral Oxygenation in Patients With Metabolic Syndrome. Front. Physiol..

[94] Broggini T., Duckworth J., Ji X., Liu R., Xia X., Mächler P., Shaked I., Munting L.P., Iyengar S., Kotlikoff M. (2024). Long-Wavelength Traveling Waves of Vasomotion Modulate the Perfusion of Cortex. Neuron.

[95] Zhang L., Wang T., Tamura Y. (2010). A Frequency–Spatial Domain Decomposition (FSDD) Method for Operational Modal Analysis. Mech. Syst. Signal Process..

[96] Kutz J.N., Brunton S.L., Brunton B.W., Proctor J.L. (2016). Dynamic Mode Decomposition.

[97] Brunton S.L., Kutz J.N. (2019). Data-Driven Science and Engineering: Machine Learning, Dynamical Systems, and Control.

[98] Kamdem C.D.B., Yomi P.A.N., Tabi C.B., Mohamadou A. (2023). Modulated Blood Waves in the Coupled Complex Ginzburg–Landau Equations of Jeffrey Fluids in Arteries. Eur. Phys. J. Plus.

[99] Cole J.D. (1977). Perturbation Methods in Fluid Mechanics (Milton Van Dyke). SIAM Rev..

[100] Fraenkel L.E. (1965). Perturbation Methods in Fluid Mechanics. By MILTON VAN DYKE. Academic Press, 1964. 229 Pp. 22 16s. J. Fluid Mech..

