# Supplementary material for: Modal Analysis of Cerebrovascular Effects for Digital Health Integration of Neurostimulation Therapies—A Review of Technology Concepts
Source: Brain Sci. 2024 Jun 10;14(6):591. doi: 10.3390/brainsci14060591 (PMC11201600; doi:10.3390/brainsci14060591)
Supplement: Supplementary file 1 [file brainsci-14-00591-s001.zip › brainsci-3031105-supplementary.pdf]

Supplementary Materials

To determine the related mode shapes following modal analysis, we need to derive the equations for blood volume waves in Jeffrey fluids from surface and penetrating arterioles to capillaries using the coupled complex Ginzburg-Landau (CGL) equations [98], we'll need to follow a sequence of mathematical and physical reasoning [99],[100]. Jeffrey fluid dynamics consider both viscosity and elasticity through the stress tensor formulation, differing from Newtonian fluids. The constitutive equation for the stress tensor (T) in a Jeffrey fluid is:

$$T = -pI + s$$

Where p is the fluid pressure, I is the identity tensor, s is the extra stress tensor related to the fluid's viscoelastic properties.

Given the shear rate (γ), the extra stress tensor (s) is defined by:

$$s = \frac{\mu}{1 + \lambda_1}(\dot{\gamma} + \lambda_2 \ddot{\gamma})$$

where μ is the viscosity, λ_1 and λ_2 are material constants representing relaxation and retardation times, $\dot{\gamma}$ and $\ddot{\gamma}$ are the first and second-time derivatives of the shear rate, respectively.

The motion of the fluid is described by the Navier-Stokes equations adapted for non-Newtonian fluids. In cylindrical coordinates (r, θ, z), and under assumptions of axisymmetric (no θ dependence) and steady state, the momentum equations simplify to:

$$\rho\left(\frac{\partial u}{\partial t} + u \cdot \nabla u\right) = -\nabla p + \nabla \cdot s + f$$

where u is the velocity vector and f represents body forces (typically gravity).

To explore wave modulation, a multiple-scale analysis is applied. This technique uses different scales for spatial and temporal variables to separate slow and fast dynamics where stretched coordinates: $(r_n = \epsilon^n r), (z_n = \epsilon^n z), (t_n = \epsilon^n t) for (n = 0,1,2, \dots)$, ϵ is a small parameter representing the perturbation level.

The physical variables (e.g., pressure p, velocity components) are expanded in power series in ϵ where $p = p_0 + \epsilon p_1 + \epsilon^2 p_2 + \cdots$. Similar expansions for velocity components and other relevant quantities are necessary. Substituting these expansions into the governing equations and equating coefficients of powers of ϵ leads to a hierarchy of equations at each order of ϵ. The leading order typically captures the primary wave dynamics, while higher orders describe modulations and interactions.

The resultant set of equations at each order can often be expressed in the form of CGL equations, which are well known in physics for describing the evolution of wave envelopes with modulational instability:

$$i\frac{\partial \psi}{\partial t} + p\frac{\partial^2 \psi}{\partial x^2} + q|\psi|^2\psi = 0$$

where ψ represents the wave envelope, p and q are coefficients derived from the fluid properties and wave characteristics (e.g., amplitude, frequency).

These equations are then solved, often numerically, to understand the conditions under which instabilities occur and how these lead to the formation of localized wave structures (solitons). Here, Jeffrey fluid dynamics can be effectively described using CGL equations, providing insights into blood wave behaviors in arteries under various

physiological and pathological conditions like vascular dementia. The CGL equation is a nonlinear partial differential equation widely used to describe complex systems near the threshold of instability of uniform states. The standard form of the CGL equation is:

$$A_t = \mu A + (1 + i\alpha)\nabla^2 A - (1 + i\beta)|A|^2 A$$

Here, A represents the complex amplitude of the wave field, A_t denotes the partial derivative of A with respect to time t, μ is a complex coefficient, α and β are real parameters controlling the dispersion and nonlinearity, respectively, and ∇^2 is the Laplacian operator.

To linearize the CGL equation, we first assume that the field A is close to often taken as zero for simplicity (e.g., small perturbations around a uniform state).

Let, $A = A_0 + a$ where A_0 is the uniform (often zero) state, and a represents a small perturbation. Plugging this into the CGL equation and assuming $A_0 = 0$ (as A_0 satisfies the equation when it's a stable solution), the terms become:

$$A_t \approx a_t$$

$$\nabla^2 A \approx \nabla^2 a$$

$$|A|^2 A \approx |A_0 + a|^2 (A_0 + a) = |a|^2 a \text{ (ignoring higher order terms)}$$

Substitute these approximations back into the CGL equation:

$$a_t = \mu a + (1 + i\alpha)\nabla^2 a - (1 + i\beta)|a|^2 a$$

To linearize, we drop the nonlinear term $(1 + i\beta)|a|^2 a$ which involves higher powers of the small perturbation a, yielding the linearized form of the CGL equation:

$$a_t = \mu a + (1 + i\alpha)\nabla^2 a$$

Analysis of the Linearized Equation: The linearized equation:

$$a_t = \mu a + (1 + i\alpha)\nabla^2 a$$

can be analyzed using methods such as Fourier transforms or by looking for solutions of the form $a(x,t) = e^{i(kx-\omega t)}$ (plane wave solutions). Here k is the wave number and ω is the frequency. This approach helps in understanding the stability and propagation characteristics of the perturbations in the medium described by the CGL equation. The linearized form of the CGL equation provides a simpler, tractable model to analyze small perturbations around a uniform state for modal analysis. Linearized form of the CGL equation is particularly useful for stability analysis and model predictive control [97], where determining whether small subthreshold tES perturbations can inform the stability of the system in health and disease for tES control. Modal decomposition can be used to identify significant characteristics of blood flows from surface to penetrating arterioles to capillaries, focusing on their energetic and dynamic aspects [48]. The modal decomposition method isolates spatial features known as modes, which are associated with specific values indicating energy levels or the rates and frequencies of growth. These modes can be extracted directly from measured flow field data or derived from the underlying mathematical equations governing the flow. Techniques that analyse flow field data are termed data-based, while those that utilize theoretical models or operators from the Navier-Stokes equations are called operator-based.
